# Supplementary material for: Empirical methods for controlling false positives and estimating confidence in ChIP-Seq peaks
Source: BMC Bioinformatics. 2008 Dec 5;9:523. doi: 10.1186/1471-2105-9-523 (PMC2628906; doi:10.1186/1471-2105-9-523)
Supplement: Additional File 1 — A variety of html documents from the USeq web site detailing the available applications, their best usage, output file type descriptions, command line menus, etc. [file 1471-2105-9-523-S1.zip › USeqUserGuides/JavaDoc.zip!/JavaDoc/edu/utah/seq/parsers/ElandParser.html]

ElandParser


|  |  |  |
| --- | --- | --- |
| **Overview** \| **Package** \| **Class** \| **Use** \| **Tree** \| **Deprecated** \| **Index** \| **Help** | |  |
| **PREV CLASS**   **NEXT CLASS** | **FRAMES**    **NO FRAMES**     **All Classes** |  |
| SUMMARY: NESTED \| FIELD \| CONSTR \| METHOD | DETAIL: FIELD \| CONSTR \| METHOD |  |


---


## edu.utah.seq.parsers Class ElandParser

```
java.lang.Object
  edu.utah.seq.parsers.ElandParser
```

---

``` public class ElandParser extends java.lang.Object ```

Parses an Eland extended \_export.txt or \_sorted.txt file into point data, split by chromosome and strand.
HWI-EAS240 FC2087UAAXX 8 1 237 408 TACACATGAATTCAACTTAAATTCCTTGTTAAAATT ZZZZZZZZZZZZZZZZZZZZZZZZZZZZZZVUUVVU chr6.fasta 147077758 F 36 73 Y
HWI-EAS240 FC2087UAAXX 8 1 311 379 TACTTGGTTAAGTAGGATGTTATTCTGCTTCTACAC ZZZZZZZZZZZZZZZZZZZZZZZZVZRZZZUVLLLL chr3.fasta 154412997 R 36 65 Y
HWI-EAS240 FC2087UAAXX 8 1 219 459 TACAACATGTACAAGCCTAAATCCTTTTAGCCAGAG ZZZZZZZZZZZZZZZZZZZZZZZZZZZZZJUOSOUQ NM Y
HWI-EAS240 FC2087UAAXX 8 1 582 773 TCATGTTAAAGTGATTGTGATGTTTGAAAACCAATG ZZZZZZZZZZZZZZZZZZZZZVZZZZJZZZSUSVVG chr6.fasta 123151134 R 36 54 Y
HWI-EAS240 FC2087UAAXX 8 1 820 387 TAAATTGTAACATAAAATTCTTATGAAATTACCTCA ZKZZZZZZZZZZZZZZZZZZZZZZIZOZZZOODOOH chr16.fasta 58060756 R 36 39 N
For each sequence a single hit is assigned to the center position of the read. Files are saved using the bar format.
Final positions are in interbase coordinates (0 start, end excluded).

**Author:**
:   david.nix@hci.utah.edu

---

**Constructor Summary**

`ElandParser(java.lang.String[] args)`


| **Method Summary** | |
| --- | --- |
| `boolean` | `loadWorkingFile()`             Splits a tag file by chromosome and strand to seperate files. |
| `static void` | `main(java.lang.String[] args)` |
| `void` | `makePointData()`             Makes the PointData hash after sorting, writes to disk. |
| `static void` | `printDocs()` |
| `void` | `processArgs(java.lang.String[] args)`             This method will process each argument and assign new varibles |

**Methods inherited from class java.lang.Object**

`equals, getClass, hashCode, notify, notifyAll, toString, wait, wait, wait`

**Constructor Detail**

### ElandParser

```
public ElandParser(java.lang.String[] args)
```


**Method Detail**

### makePointData

```
public void makePointData()
```

:   Makes the PointData hash after sorting, writes to disk.

---


### loadWorkingFile

```
public boolean loadWorkingFile()
```

:   Splits a tag file by chromosome and strand to seperate files.

---


### main

```
public static void main(java.lang.String[] args)
```

---


### processArgs

```
public void processArgs(java.lang.String[] args)
```

:   This method will process each argument and assign new varibles

---


### printDocs

```
public static void printDocs()
```


---


|  |  |  |
| --- | --- | --- |
| **Overview** \| **Package** \| **Class** \| **Use** \| **Tree** \| **Deprecated** \| **Index** \| **Help** | |  |
| **PREV CLASS**   **NEXT CLASS** | **FRAMES**    **NO FRAMES**     **All Classes** |  |
| SUMMARY: NESTED \| FIELD \| CONSTR \| METHOD | DETAIL: FIELD \| CONSTR \| METHOD |  |


---
